# Nano-Carriers Based on pH-Sensitive Star-Shaped Copolymers for Drug-Controlled Release

**DOI:** 10.3390/ma12101610

**Published:** 2019-05-16

**Authors:** Wenzhao Jiang, Jianwei Guo, Weiqiu Wen, Yong-Guang Jia, Sa Liu

**Affiliations:** 1 School of Chemical Engineering & Light Industry, Guangdong University of Technology, Guangzhou 510006, China; jwz.max@foxmail.com (W.J.); 15024028760@163.com (W.W.); 2 School of Materials Science and Engineering, South China University of Technology, Guangzhou 510641, China; ygjia@scut.edu.cn

**Keywords:** nano-carrier, pH-sensitive copolymer, drug release, adamantane

## Abstract

Polymeric nano-carriers are considered as promising tools in biomedical applications due to multiple attractive characteristics including their low toxicity, high loading capacity, controlled drug release capabilities, and highly tunable chemical properties. Here, a series of pH-sensitive star-shaped copolymers, Ad-P[(EMA-*co*-MAA)-*b*-PPEGMA]_4_, was prepared via electron transfer atom radical polymerization (ARGETE ATRP) and selective hydrolysis. These star-shaped copolymers can be self-assembled into micelles (*D*_h_ = 150–160 nm) and their critical micelle concentrations (CMC) were estimated to be 3.9–5.0 mg/L. The pH-sensitiveness of the micelles was evidenced by transmission electron microscopy (TEM) and dynamic light scattering (DLS). The maximal paclitaxel (PTX) loading efficiency (DLC) and entrapment efficiency (EE) were 18.9% and 36%, respectively. In vitro release studies revealed that about 19% of the PTX released at an acidic condition of pH 1.2 over 70 h, whereas more than 70% was released within the same time interval at pH 6.8. In vitro cytotoxicity suggested that the low cytotoxicity of the blank micelles, while the PTX-loaded micelles providing the cytotoxicity close to that of free PTX. These results indicated that this novel pH-sensitive nano-carriers have great potential applications for oral drug-controlled release.

## 1. Introduction

Polymeric nano-carriers (e.g., micelles, nanoparticles, liposomes, etc.) have been attracted considerable interest due to low toxicity, drug-controlled release abilities, high drug efficacy, et al. [1,2,3,4,5,6,7,8]. Especially, pH-sensitive nano-carriers have remarkable properties which enable them to bypass biological barriers and reach the goal of targeted drug delivery [9,10]. Poly(methacrylic acid) (PMAA), bearing the carboxylic group with a pKa around 5–6, is frequently used in pH-sensitive polymers [11]. The block PMAA of copolymers could keep tight in the protonated form under an acidic environment, whereas in the basic condition, the deprotonation and ionization of PMAA contribute to the swell of the blocks. Such pH-sensitive properties can be used as oral drug delivery systems, where the drug could go through the acidic environment of stomach and be released upon increasing the pH. For instance, Yang et al. prepared a series of a linear amphiphilic copolymer containing a PMAA block for oral hydrophobic drug delivery [12,13,14].

However, compared with traditional linear polymers, star-shaped copolymers present unique properties and advantages, such as multiple topological structures, increased stability, high functionality, and also have attracted extensive attention [15,16]. For example, Zhang’s group synthesized a series of tetramethylolmethane-based or β-cyclodextrin-based polymeric micelles for drug/DNA delivery [17,18,19]. Zhu et al. made an in-depth study on cholic acid-based star polymers for drug delivery system [20,21,22]. Recently, star-shaped copolymers bearing rigid molecular core (e.g., POSS) use as nano-carriers of drugs or DNA have attracted more attention [23,24,25]. Adamantane (Ad) with the same rigid and cubic core as POSS, but the good lipophilicity and biocompatibility, has been widely studied in medicine, functional materials as well as nanotechnology. Owing to their biocompatibility, low-toxicity and facile accessibility, Ad should be regarded as a promising scaffold for drug delivery systems [26,27,28]. For instance, Guo et al. developed a series of pH-responsive star-shaped polymer Ad-(PCL-*b*-PDEAEMA-*b*-PPEGMA)_4_. The micelles prepared from these copolymers released 67% of doxorubicin (DOX) at pH 4.5 due to the protonation of block poly(*N,N*-diethylamino-2-ethylmethacrylate) (PDEAEMA), while only 20% of DOX was released at the pH 7.4. This fast release behavior driven by the tertiary amine groups under the acidic conditions is not suited for use as oral drug delivery nano-carriers [29]. Therefore, it is highly demanded to explore novel pH-sensitive nano-carriers assembled by Ad-based star-shaped polymers for oral drug delivery.

**Scheme 1:**
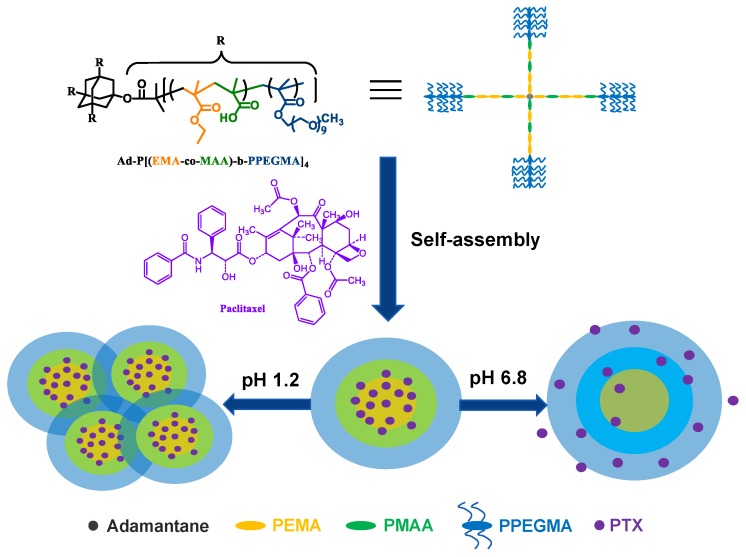
Schematic formation of nano-carriers and the illustration of pH-dependent drug release.

Herein, pH-sensitive nano-carriers based on star-shaped copolymer, adamantane-poly[(ethylmethacrylate-*co*-methacrylic acid)-*b*-poly(poly(ethyleneglycol)methyl ether methacrylate)]_4_ (Ad-P[(EMA-*co*-MAA)-*b*-PPEGMA]_4_), were designed for oral drug controlled release. The hydrophobic PEMA was incorporated randomly with the pH-sensitive PMAA, forming the core of micelles and rendering the pH-sensitiveness of the micelles. The hydrophilic block PPEGMA could form the out layer of micelles, for maintaining the stability and increasing water solubility. Paclitaxel (PTX), one of the hydrophobic anticancer drug, was used as a model drug and encapsulated into the Ad-P[(EMA-*co*-MAA)-*b*-PPEGMA]_4_ micelles. Such PTX-loaded micelles could keep a tight structure in an acidic environment (pH 1.2). The deprotonation of -COOH groups of PMAA at pH 6.8 may contribute to the swell of the micelle structure, thus the PTX could be released (Scheme 1). The cytotoxicity of these nano-carriers was also studied.

## 2. Materials and Methods

### 2.1. Materials

Ethyl methacrylate (EMA), *tert*-butyl methacrylate (*t*BMA), poly(ethylene glycol) methyl ether methacrylate (PEGMA, *M*_n_ = 500 Da), *N,N,N′,N′,N″*-pentamethyldiethylenetriamine (PMDETA), CuBr_2_, stannous octoate (Sn(Oct)_2_), trifluoroacetic acid (TFA), pyrene and paclitaxel (PTX) were purchased from Aladdin (Aladdin Chemistry Co., Ltd., Shanghai, China).

### 2.2. Characterization

^1^H NMR spectra were recorded on Bruker AVANCE Ш 400 MHz (Bruker, Rheinstetten, Germany) using deuterated chloroform (CDCl_3_-*d*) or sulfoxide-*d*_6_ (DMSO-*d*_6_) as a solvent. The number average molecular weight (*M*_n_) and dispersity index (*M*_w_/*M*_n_) were measured on a gel permeation chromatography (GPC) using Waters 1525/2414 (Waters, Milford, MA, USA) with THF as the eluent (1.0 mL/min). The particle size (*D*_h_) and zeta potentials were determined by dynamic light scattering (DLS) with Brookhaven (Brookhaven, NY, USA). Transmission electron microscopy (TEM, HITACHI HT7700, Tokyo, Japan) was employed to characterize the morphologies of the nanoparticles. Fluorescence spectra were obtained using a fluorescence spectrophotometer (FluoroMax-4, HORIBA Jobin Yvon, Edison, NJ, USA).

### 2.3. Synthesis of Ad-P[(EMA-co-MAA)-b-PPEGMA]_4_

#### 2.3.1. Synthesis of Ad-P[(EMA-*co*-tBMA)]_4_

Ad-P[(EMA-*co*-*t*BMA)]_4_ was synthesized by ARGET ATRP of EMA and *t*BMA. In brief, Ad-(Br)_4_ (0.12 g) (Figure S1) [30], EMA (3.3 mL, 26.5 mmol), *t*BMA (3.4 mL, 21 mmol), CuBr_2_ (11.2 mg, 0.05 mmol), PMDETA (105 μL, 0.5 mmol), Sn(Oct)_2_ (160 μL, 0.5 mmol) and 30 mL of THF were placed in a 100 mL Schlenk flask and degassed by three-freeze-thaw cycles. The mixture was then heated to 60 °C for 24 h under an argon atmosphere. After cooling to room temperature, the catalyst was removed by passing through a neutral alumina column (THF as the eluent). Finally, after precipitating by n-hexane, the final product was dried under vacuum for 24 h.

#### 2.3.2. Synthesis of Ad-P[(EMA-*co*-tBMA)-*b*-PPEGMA]_4_

The synthetic procedure of Ad-P[(EMA-*co*-*t*BMA)-*b*-PPEGMA]_4_ was similar to Ad-P[(EMA-*co*-*t*BMA)]_4_. In brief, Ad-P[(EMA-*co*-*t*BMA)]_4_ (0.5 g), PEGMA (3.5 mL, 7.7 mmol), CuBr_2_ (11.2 mg, 0.05 mmol), PMDETA (105 μL, 0.5 mmol), Sn(Oct)_2_ (160 μL, 0.5 mmol) and 30 mL of THF were placed in a 100 mL Schlenk flask and degassed by three-freeze-thaw cycles. The mixture was then heated to 60 °C for 24 h under Argon atmosphere. After cooling to room temperature, the catalyst was removed by passing through a neutral alumina column (THF as the eluent). Finally, after precipitating by n-hexane, the final product was dried under vacuum for 24 h.

#### 2.3.3. Hydrolysis of Ad-P[(EMA-*co*-tBMA)-*b*-PPEGMA]_4_

Ad-P[(EMA-*co*-MMA)-*b*-PPEGMA]_4_ was synthesized by hydrolysis. Briefly, Ad-P[(EMA-*co*-*t*BMA)-*b*-PPEGMA]_4_ (0.5 g) was dissolved in 20 mL of DCM. 2 mL of TFA was added slowly into the mixture with vigorous stirring under 0 °C. After stirring at 0 °C for 30 min, the reaction was carried out at 25 °C for 24 h. After evaporating all the solvent, the residues were dissolved in 5 mL of THF and precipitated into 50 mL of n-hexane. The resulting copolymer Ad-P[(EMA-*co*-MMA)-*b*-PPEGMA]_4_ was collected by filtration and dried under vacuum for 24 h.

### 2.4. Critical Micelle Concentration (CMC) Measurement

The CMC of the copolymer was measured using pyrene as a hydrophobic fluorescent probe The copolymer solutions in various concentrations were equilibrated with a constant concentration of pyrene (6 × 10^−7^ M) for 24 h in the dark. The CMC values were determined by the fluorescence excitation spectra.

### 2.5. Study of the pH-Sensitive of the Blank Micelles

In brief, 40 mg of the copolymer was dissolved in 10 mL of DMF. Under stirring, the solution was added dropwise to deionized water (40 mL). The mixture was placed into a dialysis bag (Molecular weight cut off (MWCO) = 3.5 kDa) and dialyzed against deionized water for 48 h at 25 °C. The blank micelle solution was further used to study the pH-sensitiveness. Acid-base titration was measured (the method was shown in the Supplementary Materials). Dynamic light scattering (DLS) and transmission electron microscopy (TEM) were used to test the particle size, zeta potential and morphologies of the nano-carriers in different pHs.

### 2.6. Preparation of PTX-Loaded Micelles

In brief, 40 mg of copolymers and 13 mg of PTX were dissolved in 10 mL of N,N-dimethylformamide (DMF). The solution was transferred into a dialysis bag (MWCO = 3.5 kDa) and dialyzed against deionized water for 48 h at 25 °C. Finally, the PTX-loaded polymers were collected by lyophilization. The drug loading content (DLC) and entrapment efficiency (EE) were calculated using formula:

(1) $$\mathrm{DLC}\;(\%)=\frac{m_{loaded\;drug}}{m_{drug-loaded\;micelle}}\times 100\%$$

(2) $$\mathrm{EE}\;\left(\%\right)=\frac{m_{loaded\;drug}}{m_{drug\;in\;feed}}\times 100\%.$$

### 2.7. In Vitro PTX Release Study

In brief, PTX-loaded micelles (5 mg) were suspended in 5 mL of simulated gastric fluid (SGF, pH 1.2) or simulated intestinal fluid (SIF, pH 6.8), and then placed into a dialysis bag (MWCO = 3.5 kDa). The bag was placed into 35 mL of SGF or SIF solution. Sample solution (4 mL) was taken out at specified time intervals and 4 mL of fresh SIF or SGF solution was added to maintain the total volume at 37 °C. The cummulative PTX release percent (*E_r_*) was calculated using formula:

(3) $$E_{r}(\%)=\frac{V_{e}\sum_{1}^{n-1}C_{i}+V_{0}C_{n}}{m_{PTX}}\times 100$$

where *m*_PTX_ is the amount of PTX in the micelles, *V*_0_ = 40 mL, *C_i_* is the concentration of PTX in the ith sample. Each experiment was repeated three times.

### 2.8. In Vitro Cytotoxicity Test

The in vitro cytotoxicity of the blank and PTX-loaded micelles were evaluated against 3T3 and MCF-7 cells by the CKK-8 assay. The 3T3 and MCF-7 cells were first cultured and maintained in DMEM. Cells seeded in 96-well plates were incubated in 5% CO_2_ at 37 °C with blank and PTX-loaded micelle solution with different concentration for 48 h. Afterwards, 10 μL of freshly prepared CKK-8 solution was added to each well and incubation for another 2 h. The absorbance was measured at 450 nm by a microplate reader. Cell viability was expressed as the percentage of surviving cells compared to the cells in untreated control wells.

## 3. Results and Discussion

### 3.1. Synthesis and Characterization of Ad-P[(EMA-co-MAA)-b-PPEGMA]_4_

**Scheme 2:**
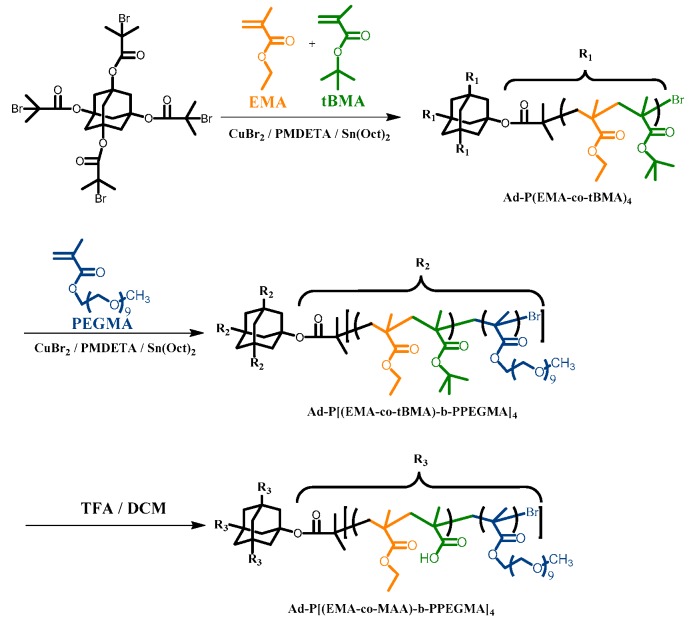
Synthetic route of Ad-P[(EMA-*co*-MMA)-*b*-PPEGMA]_4_.

**Figure 1:**
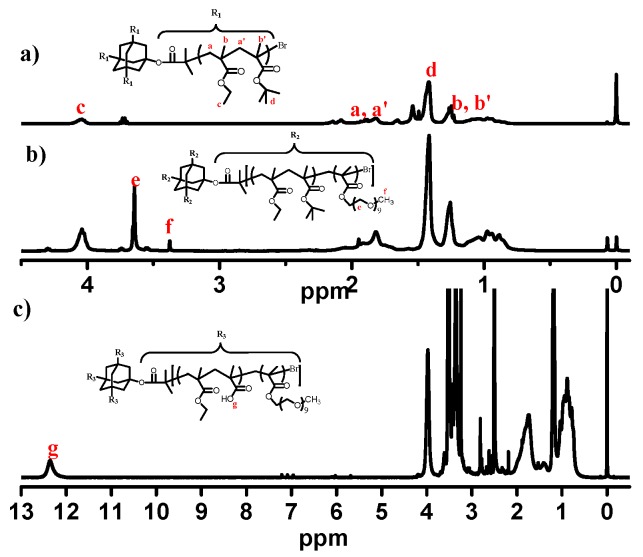
Proton nuclear magnetic resonance (^1^H NMR) spectra of Ad-P[(EMA-*co*-*t*BMA)]_4_ in CDCl_3_-*d* (**a**); Ad-P[(EMA-*co*-*t*BMA)-*b*-PPEGMA]_4_ in CDCl_3_-*d* (**b**); Ad-P[(EMA-*co*-MAA)-*b*-PPEGMA]_4_ in DMSO-*d*_6_ (**c**).

**Figure 2:**
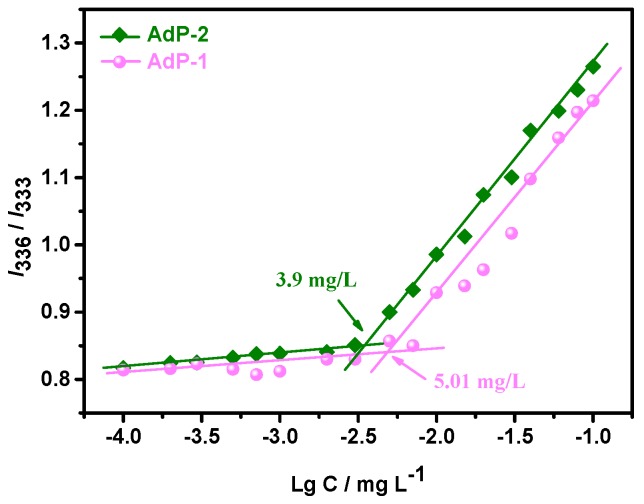
Critical micelle concentration (CMC) values of AdP-1 and AdP-2.

Ad-P[(EMA-*co*-MAA)-*b*-PPEGMA]_4_ was synthesized via ARGET ATRP and selective hydrolysis of *tert*-butyl groups of *t*BMA, as shown in Scheme 2. The signals of -CH_2_- and -CCH_3_ on methacrylate backbone appear at 1.8–2.0 ppm (a, a′) and 0.9–1.2 ppm (b, b′), respectively, on ^1^H NMR spectra in Figure 1a. Meanwhile, the peaks at 4.05 ppm (c) and 1.42 ppm (d) are ascribed to the -CH_3_ on EMA unit and -C(CH_3_)_3_ on *t*BMA unit, respectively. The peaks at 3.38 (f) and 3.64 (e) ppm were assigned to the signals of -OCH_3_ and -OCH_2_CH_2_- protons in the PEGMA unit (Figure 1b). As seen in Figure 1c, after hydrolysis, the *tert*-butyl groups (1.42 ppm) disappears, while the signal of the carboxyl group can be observed at 12.37 ppm (g). According to GPC analysis, the *M*_n_ of the copolymers Ad-P1 and Ad-P2 were about 21.4, and 20.9 × 10^3^ Da with the *M*_w_/*M*_n_ of 1.33, and 1.5, respectively (Table 1, Figure S2). In addition, the CMC values of AdP-1 and AdP-2 were determined to be ~5.0 and 3.9 mg/L, respectively (Figure 2 and Table 1). The low CMC values indicated that stable micelles could be formed at a lower concentration, and may endow the carriers with long-circulating characteristic [31].

### 3.2. pH-Sensitive Behaviors of Blank Micelles

**Figure 3:**
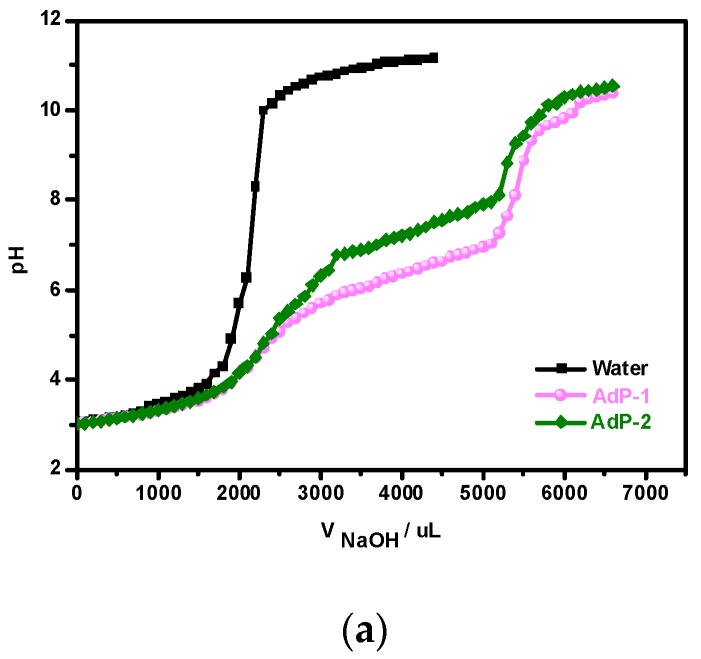

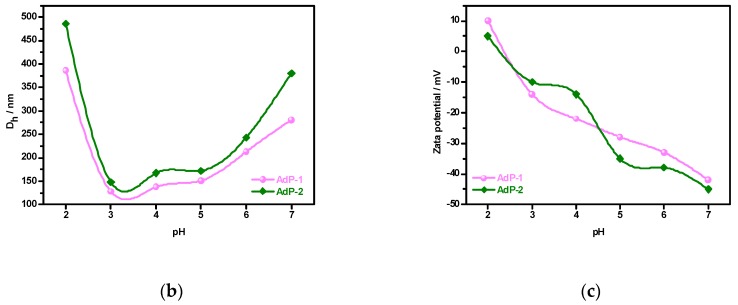
Titration curves (**a**); Effects of pH on *D*_h_ (**b**) and zeta potential (**c**) of the micelles formed by Ad-P1 and AdP-2.

**Figure 4:**
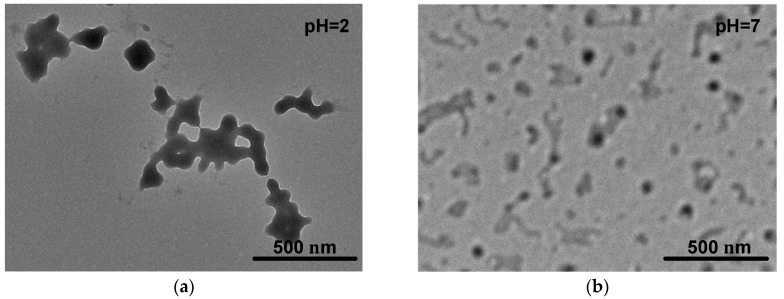
Transmission electron microscope (TEM) images of blank micelles formed by Ad-P1 at different pH conditions (**a**) pH = 2; (**b**) pH = 7.

The pH-sensitive ranges of AdP-1 and AdP-2 were evaluated by the acid-base titration, as shown in Figure 3a. With the increase of NaOH solution, the pH values increased gradually and reached a plateau, because of the deprotonation of carboxyl groups (-COOH) in PMAA segments. The buffering pH regions of AdP-1 and AdP-2 were 5.2–8.1. It also could be seen that AdP-2 with longer PMAA segments needs more NaOH solution. Furthermore, the particle size (*D*_h_), zeta potential and morphologies of blank micelles at different pHs were measured to investigate the pH-sensitiveness. In Figure 3b (Figure S3), the particle size is larger than 400 nm at pH 1–2, probably due to the aggregation of the micelles formed by the hydrogen bonding from -COOH of PMAA. At pH = 3–5, the size of the micelle was kept below 200 nm, indicating the stability of the micelle structure [12,32,33]. In the pH range of 5–7, the size of the micelles showed an increasing trend, which may be related to the deprotonation of PMAA and swelling of the micelles. Zeta potential presented higher negative charges as shown in Figure 3c also confirmed the ionization of the -COOH groups from PMAA [24]. The negative charges of the micelles could benefit the bioadhesion between the micelles and intestinal epithelial cells [12,34,35,36]. TEM images in Figure 4 reflected the morphologies of the micelles in different pHs. At pH 2, the micelles may congregate into irregular aggregates due to hydrogen bonding among micelles. Due to the deprotonation at pH 7, the swelling of micelles may lead to some destruction of the micellar structures.

### 3.3. In Vitro PTX Controlled Release

**Figure 5:**
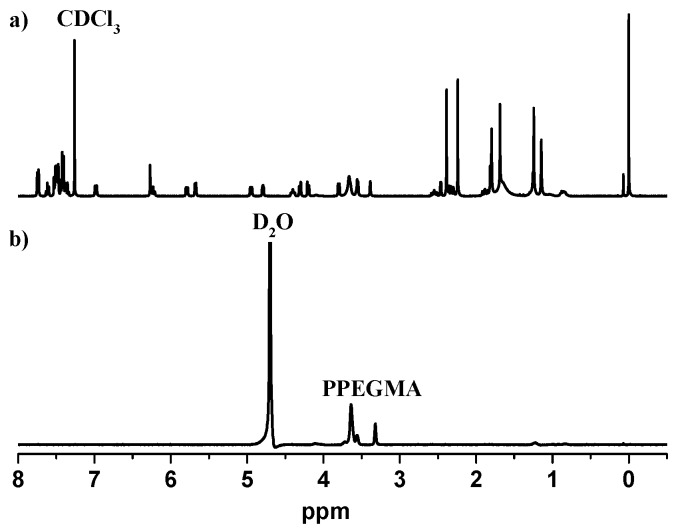
^1^H NMR spectra of PTX-loaded micelles in CDCl_3_-*d* (**a**); PTX-loaded micelles in D_2_O (**b**).

**Figure 6:**
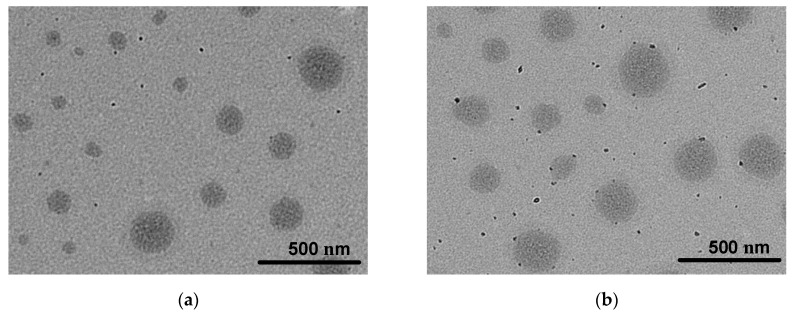

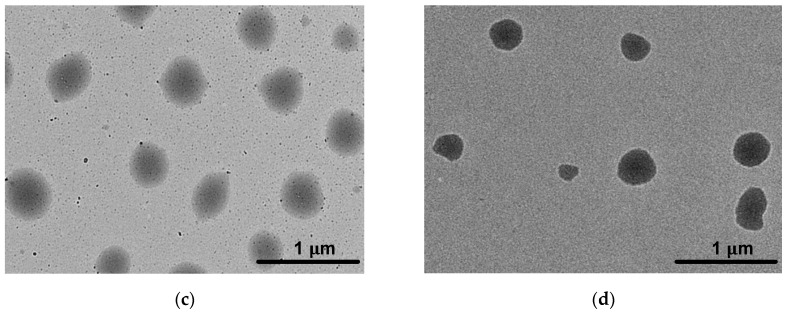
TEM images of AdP-1 blank micelles (**a**), AdP-2 blank micelles (**b**), AdP-1 PTX-loaded micelles (**c**) and AdP-2 PTX-loaded miceles (**d**).

As shown in Figure 5, because chloroform is a good solvent for both PTX and polymer, so the signals of PTX and the polymeric micelles have been observed (Figure 5a). In D_2_O, only signals of PPEGMA are visible whereas the signals corresponding to PTX and hydrophobic segments disappear (Figure 5b). This indicates that the PTX was encapsulated into the core of the polymeric micelles. The size of blank micelles was about 150–160 nm and increased to be 300 nm after loading PTX (Table 2). Drug loading content (DLC) and entrapment efficiency (EE) were influenced by the weight ratio of drug to copolymer [13,29,37]. A series of PTX-loaded micelles with different weight ratios of copolymers were optimized. As the amount of PTX increased, the DLC was enhanced gradually, while the EE increased first and then decreased. The results showed that the optimal drug loading efficiency was obtained at a ratio of PTX and copolymer 13:40 (≈1:3). According to previous studies, the micelles with longer hydrophobic segment have higher drug loading content and entrapment efficiency [29,38,39]. Under the same drug feed ratio test condition, AdP-2 exhibited higher drug entrapping ability than AdP-1, due to the longer hydrophobic content of AdP-2. Both blank and PTX-loaded nano-carriers showed spherical morphology on TEM images (Figure 6).

**Figure 7:**
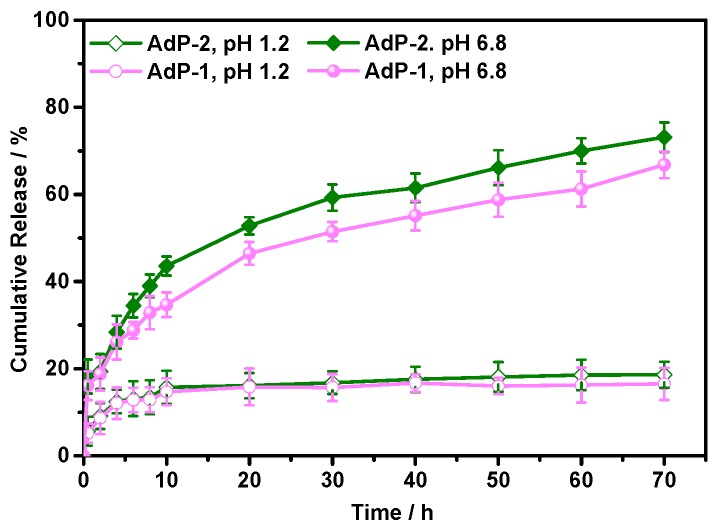
In vitro release profiles of PTX-loaded micelles.

In vitro release profiles of PTX-loaded micelles nano-carriers were measured at 37 °C in SGF (pH 1.2) and SIF (pH 6.8), respectively (Figure 7). At pH 1.2, due to the tight structure of PTX-loaded micelles, the release rates were slow and only about 19% of PTX was released after 70 h, indicating the PTX can be protected in the acidic environment. At pH 6.8, the release rates of PTX were accelerated without burst release and 34–44% of PTX released in 10 h and 67–73% in 70 h, respectively, due to the swelling of micelles caused by the -COOH groups of PMAA. As PEMA and PMAA were defined as a random copolymer, the pH-sensitiveness of the micelles increased with the increase of PMAA ratios. Thus, at pH 6.8, the cumulative release of AdP-2 (73%) was higher than that of AdP-1 (67%). In brief, the in vitro PTX release tests demonstrated that the pH-sensitive micelles can be potentially used as oral drugs nano-carriers. In addition, to evaluate the mechanism of drug release from nano-carriers, release data were fitted with zero order, first order, Hixson-Crowell and Korsmeyer-Peppas kinetic models, respectively [40,41,42,43] in Table 3. The Hixson-Crowell model may be the best-fitted kinetic model.

### 3.4. In Vitro Cytotoxicity Test

**Figure 8:**
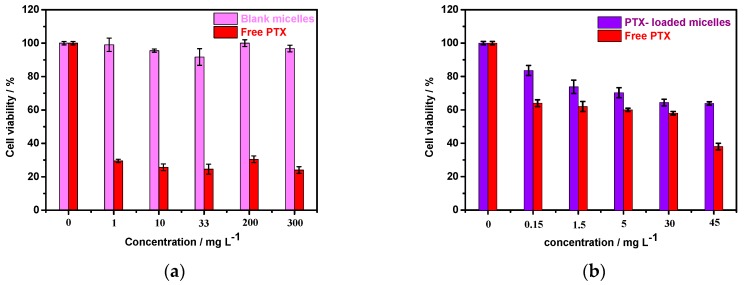
Cytotoxicity of blank and PTX-loaded micelles against 3T3 cells (**a**) and MCF-7 cells (**b**) for 48 h.

For the potential biomedical applications, it is necessary to investigate the cytotoxicity of polymeric nano-carriers. Figure 8 showed the results of cells treated with blank and PTX-loaded micelles for 48 h, respectively. It should be noticed that the viability of the 3T3 cells was observed to be close to ~90% even at higher concentration of 300 mg/L after 48 h (Figure 8a), indicating the good cytocompatibility of nano-carriers. In addition, PTX-loaded micelles or PTX was used in the incubation of MCF-7 cells and both of them showed significant antitumor activity, which positively correlated with their concentrations (Figure 8b).

## 4. Conclusions

Star-shaped pH-sensitive copolymers Ad-P[(EMA-*co*-MMA)-*b*-PPEGMA]_4_ were synthesized by ARGET ATRP. The copolymers can be self-assembled into micelles with pH-sensitiveness. In vitro drug release behaviors of the PTX-loaded micelles suggested that the rate of the PTX release could be controlled by the pH values of the environment. Less than 20% of PTX released in SGF solution (pH 1.2), while 67–73% of PTX released in SIF solution (pH 6.8) within 70 h. The in vitro cytotoxicity assay confirmed the low cytotoxicity of the blank micelles formed by star-shaped copolymers. Thus, the as-made pH-sensitive micelles could be the promising oral drug nano-carriers for biomedical applications. In addition, further and more studies (e.g., cell uptake test and in vivo test, such as cytotoxicity, biocompatibility, and in vivo drug release) is ongoing in our lab.

## Figures and Tables

**Table 1:** Characterization of Ad-P[(EMA-*co*-MMA)-*b*-PPEGMA]_4_.

	Sample	*M*_n, GPC_^1^	*M*_w_/*M*_n_^1^	*M*_n, th_^2^	CMC ^3^ (mg/L)
AdP-1	Ad-P[(EMA_11_-*co*-MAA_9_)-*b*-PPEGMA_10_]_4_	21.4 × 10^3^	1.33	25.1 × 10^3^	5.0
AdP-2	Ad-P[(EMA_11_-*co*-MAA_12_)-*b*-PPEGMA_7_]_4_	20.9 × 10^3^	1.50	23.5 × 10^3^	3.9

^1^ Determined by GPC. ^2^ Value by design. ^3^ Calculated from a fluorescence spectroscopic method.

**Table 2:** The properties of blank and paclitaxel (PTX)-loaded micelles.

	PTX/Copolymer (mg/mg)	*D*_h_ (nm)	PDI	Zata Potential (mV)	DLC (%)	EE (%)
AdP-1	0/40	150.8	0.155	−28	–	–
	10/40	196.1	0.227	−23	11.8	32.9
	13/40	265.2	0.233	−18.6	14.6	33.3
	20/40	294.8	0.248	−25	15.9	26.1
AdP-2	0/40	160.2	0.183	−30	–	–
	10/40	258	0.197	−26.8	14.7	38.9
	13/40	300.6	0.258	−21.3	18.9	36
	20/40	330	0.210	−24.1	16.8	35.7

**Table 3:** In vitro release kinetics equation simulation of the nano-carriers.

Model	Equation	*R* ^2^	*R*
**AdP-1 at pH 1.2**			
Zero order	Y = 0.1048x + 10.979	0.5189	0.7203
First order	Y = 10.38e^0.009x^	0.4083	0.6390
Hixson-Crowell	Y = 0.0002x^3^ − 0.0224x^2^ + 0.8466x + 7.2662	0.8838	0.9401
Peppas	Y = 7.7009x^0.2103^	0.8609	0.9278
**AdP-2 at pH 1.2**			
Zero order	Y = 0.1328x + 11.281	0.6486	0.8053
First order	Y = 10.77e^0.01x^	0.5034	0.7100
Hixson-Crowell	Y = 0.0002x^3^ − 0.0205x^2^ + 0.8156x + 7.8419	0.8949	0.9460
Peppas	Y = 8.009x^0.2215^	0.9159	0.9570
**AdP-1 at pH 6.8**			
Zero order	Y = 0.6742x + 24.549	0.9045	0.9511
First order	Y = 24.52e^0.017x^	0.7829	0.8848
Hixson-Crowell	Y = 0.0004x^3^ − 0.0481x^2^ + 2.3484x + 15.822	0.9968	0.9984
Peppas	Y = 17.443x^0.3092^	0.9811	0.9905
**AdP-2 at pH 6.8**			
Zero order	Y = 0.7379x + 28.67	0.8665	0.9310
First order	Y = 28.054e^0.016^	0.7291	0.8539
Hixson-Crowell	Y = 0.0005x^3^ − 0.0625x^2^ + 2.9045x + 17.412	0.9883	0.9941
Peppas	Y = 19.648x^0.3128^	0.9651	0.9824

## Supplementary Materials

The following are available online at https://www.mdpi.com/1996-1944/12/10/1610/s1, Figure S1: ^1^H NMR spectrum of Ad-(Br)_4_, Figure S2: GPC traces of AdP-1 and AdP-2, Figure S3: The DLS data of AdP-1 and AdP-2 in different pH values.
