# Supplementary material for: Nano-Carriers Based on pH-Sensitive Star-Shaped Copolymers for Drug-Controlled Release
Source: Materials (Basel). 2019 May 16;12(10):1610. doi: 10.3390/ma12101610 (PMC6566147; doi:10.3390/ma12101610)
Supplement: Supplementary file 1 [file materials-12-01610-s001.pdf]

materials

Supporting Information

Nano-Carriers Based on pH-Sensitive Star-Shaped Copolymers for Drug-Controlled Release

Wenzhao Jiang [1], Jianwei Guo [1,*], Weiqiu Wen [1], Yong-Guang Jia [2] and Sa Liu [2,*]

1 School of Chemical Engineering & Light Industry, Guangdong University of Technology, Guangzhou 510006, China; jwz.max@foxmail.com (W.J.); 15024028760@163.com (W.W.)
2 School of Materials Science and Engineering, South China University of Technology, Guangzhou 510641, China; ygjia@scut.edu.cn
* Correspondence: guojw@gdut.edu.cn (J.G.); sliu@scut.edu.cn (S.L.)

Synthesis of Ad-(Br)$_4$

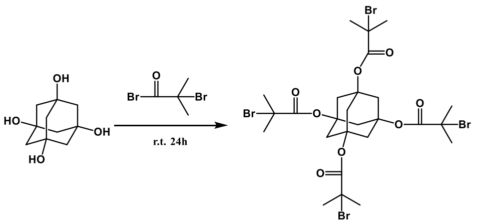

Ad-(OH)$_4$ [1] (1 g, 1.29 mmol) and pyridine (0.4 g, 5.16 mmol) were dissolved in 15 mL of acetonitrile. After cooling to 0°C with ice bath, α-bromoisobutyryl bromide (1.18 g, 5.16 mmol) was added slowly with vigorous stirring. The mixture was stirred at 0℃ for 30 min and then at room temperature for 24 h. After evaporating all the solvent, the residues were dissolved in ethyl acetate. After washing with DI water three times, the organic layer was collected and precipitated into alcohol. The resulting product was collected by filtration and dried under 40 ℃ for 24 h.

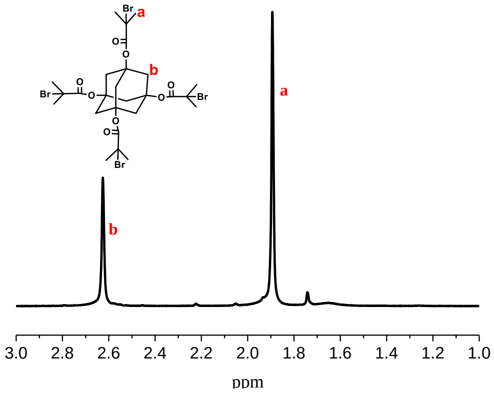

Figure S1. ^{1}H NMR spectrum of Ad-(Br)$_4$.

Acid-base titration

The copolymer was dissolved in deionized water with concentration of 1 mg/mL and adjusted the pH to 3 by 0.1 M HCl solution. Then the solution was titrated by 0.1 M NaOH solution at increment of 50 μL. The real-time pH values were recorded by automatic titrator (Hanon T-860, Jinan, China).

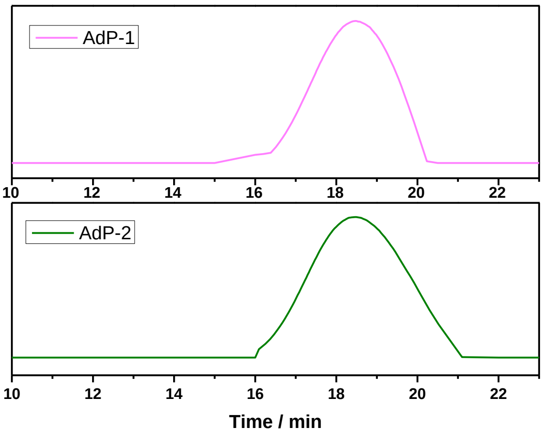

Figure S2. GPC traces of AdP-1 and AdP-2.

AdP-1 DLS

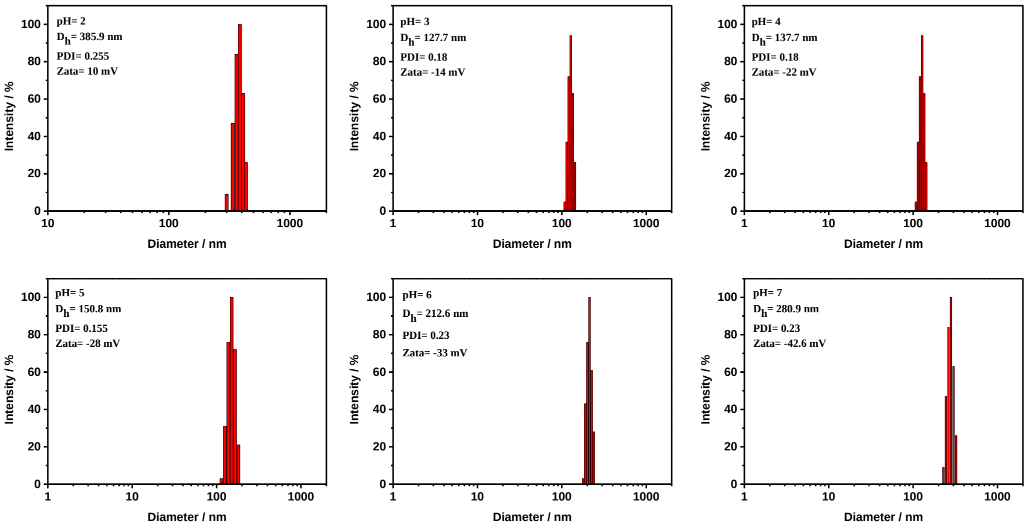

(**a**)

AdP-2 DLS

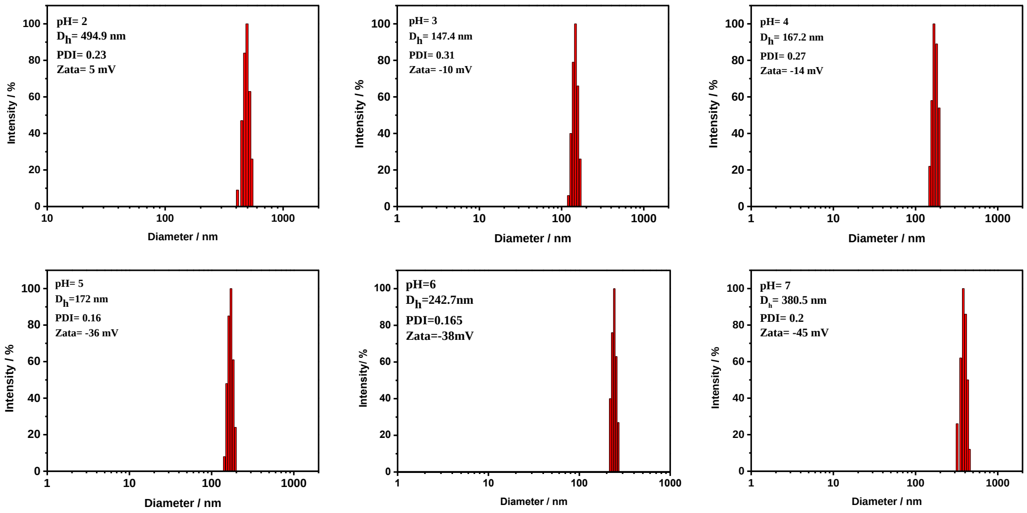

(**b**)

Figure S3. The DLS data of AdP-1 (**a**) and AdP-2 (**b**) in different pH values.

References

1. Fu, S.Q.; Guo, J.W.; Zhu, D.Y.; Yang, Z.; Yang, C.F.; Xian, J.X.; Li, X. Novel halogen-free flame retardants based on adamantane for polycarbonate. *Rsc Adv.* **2015**, *5*, 67054-67065, 10.1039/c5ra10887j.
